# On Specialism; and on the Influence of Medical Science on Modern Civilization

**Published:** 1882-01

**Authors:** Edwin Saunders

**Affiliations:** London


THE DENTAL REGISTER.

VOL. XXXVI.] JANUARY, 1882. [No. 1.

### ON SPECIALISM; AND ON THE INFLUENCE OF MEDICAL SCIENCE ON MODERN CIVILIZATION.

BY EDWIN SAUNDERS, ESQ., F. R. C. S., LONDON.

SURGEON DENTIST TO THE QUEEN.

It is useless to disguise the fact that the splitting up of Medical and Surgical practice into departments, or specialties, has always been depreciated and discouraged by those corporate bodies which charge themselves with the maintenance of the educational standard, the honour, and the public reputation of the profession. And a strong case must be made out for a departure from this rule, before such a proposition can have any chance of receiving the seal of official sanction. And the reasons for this jealousy of the disintegration of the profession are not far to seek. First amongst these is probably the apprehension that the educational standard might thereby be lowered; the limitation of the area in that wondrous microcosm, the human body which falls under his charge, having a tendency to set bounds to biological research, and general scientific attainment, on the part of the specialist. Moreover, if the department of practice is one which involves, in its successful prosecution, large and constant demands of time, as well as physical fatigue, it must inevitably result that his mental vision will gradually become myophic, that he will begin to find himself trammeled by the routine of his daily work, and indisposed by his exhausted energies, from disporting himself in "those fresh fields and pastures new," which were the charm and the dream of his student days. In fact, the specialist is not long in waking up to the consciousness that he has, by devoting himself to one particular kind of practice, set limits to his intellectual horizon, and done much to crush out any poetical aspirations which may have consciously been lying dormant in his soul. He has, indeed, become like those unfortunate birds, kept for the exercise and display of man's skill, whose wings, being clipped, become thereby disqualified for that higher range of flight, which by natural structure and instinct they are otherwise fitted to enjoy. Another reason against specialism (though this is rather subjective, and is liable to the modifications of temperament and mental idiosyncrasy), is that, being habitually deferred to in his own special department, he is in danger of forming an exaggerated estimate of his value and importance in the social scale. To counteract any tendency towards this latter infirmity, there is no readier or more certain means than to become enrolled in associations such as that with which it is our privilege to be connected, where, measuring swords in friendly conflict, we may get to know where our real strength or weakness may lie. But after all has been said that can be urged, and all objections and arguments to the contrary, notwithstanding, it is extremely improbable that special practice can ever be set aside. It is a tree of great age. having its roots in a remote antiquity, and still, in these modern days of the ninteenth century, pushing forth branches of undeniable vigor and and beauty. In fact, it must be regarded as one of those natural products, which it is equally futile and impolitic to ignore, and which persists in asserting itself as the legitimate outcome of the scientific growth and advanced civilization of the age. How constantly do those who are most strenuous in their denunciations of specialism, resort in their own case, or in that of those in whom they are nearly interested, to those who (themselves sternly repudiating the name of specialist) are looked upon as the highest authority as regards the particular ailment in question. Do we not all in similar circumstances act on one unvarying principle? In lung disease does not the name of A. rise to our lips? For heart disease do we not go in quest of B., in epilepsy for C., in

Bright's disease or gout or jaundice, is it not our first impulse to enquire whose opinion is of the greatest value, as having had the largest experience and paid the closest attention to the symptoms and treatment of these cases respectively? And if this be so in medicine, much more is it the case with all that large class of ailments in which manipulative treatment and dexterity are necessary for their relief or cure. Here again we find theory and practice in opposition, for though we are bound to regard every qualified surgeon as duly equipped and accomplished for the performance of any operation whatsoever, who, amongst us, I would ask, would not rather have a voice in the selection of the operator in a case of cataract, or in passing the catheter? For there are hands and hands, some with fingers so well formed and endowed with so exquisite a sensibility as to be entitled to be called intelligent fingers, which seem to bring the inert material instrument they grasp into direct communication with the sensorium, and some so irresponsive to the will, and altogether so intractable as to debar the possessor from doing justice to his own attainments. And permit me to add that knowing, as I do, what, as Dr. Johnson might have said, the potentiality of pain is in a carious tooth, I should most respectfully but most unhesitatingly decline the services of the most accomplished Member of the Royal College of Surgeons who had had no special training in the treatment of those organs.

And this avowal strikes the keynote of the present position of Dental Surgery, and furnishes the occasion of an apology or a statement of the *raison d'etre* of a special diploma and a distinct register for that branch of surgery. In what has been advanced with respect to the prevalence of special practice, and in the cases which have been adduced with the view of showing that it is inherent in the complex civilization of the present advanced state of society; no such sharp line of demarcation has been presented as to require a distinct curriculum or a separate examination. And for this simple and obvious reason that the structures concerned are for the most part homologous, subject to the same physiological laws, amenable to similar treatment, and possessing considerable reparative or even reproductive power, the soft parts being abundantly supplied with vessels and nerves, and the bones being protected by membrane or integument enjoying vitality of a high order. But the practitioner of Dental Surgery, having charge of organs possessed of great density, and encased in an envelope of crystalline hardness, of so low an organziation as to be at the mercy of chemical action or of mechanical lesion, and being extruded and exposed except at their articulating processes or roots, is under the necessity of employing a totally different class of instruments with quite other modes of operative procedure. This is not the time or place to open up the much vexed question of the nature and causes of decay of the teeth; for our present purpose, it will be sufficient to regard it as the result of chemical action, inasmuch as the invaluable operation known by the name of stopping, proceeds upon that assumption. A small fissure is observed in the grinding surface or other parts of an otherwise healthy tooth, arising, it may be, from imperfect apposition of the enamel fibres, sufficient to allow of the infiltration of the secretions of the mouth with more or less debris of food. It is also not improbable that the subjacent dentine is imperfect, in which case the mischief will make rapid progress, the conditions favorable to the destruction of tissue by chemical action being present in the small external opening of the cavity, and the difficulty of removing the putrescent matters within. If this is allowed to go on unchecked, the walls of the pulp cavity being weakened or destroyed on the side of the lesion, the pulp becomes exposed; and it is fortunate if at this stage the most excruciating pain to which human nature is liable is not experienced. It is the business of the Dental Surgeon to prevent this, and to restore the damaged organ to health and efficiency; but however diligent he may have been at lecture, and however observant in the wards of the hospital, his surgical knowledge will stand him in little stead, and he must seek elsewhere than in the Armamentarium Chirurgicum for the appliances proper to the case before him. He will first proceed to enlarge by a drill or strong cutting instrument the external opening, so that no enamel fibres shall be left which are unsupported by sound dentine, and he will then proceed to cut away the carious bone with small delicate chisels, straight or bent at various angels according to the position of the cavity. He will then by careful packing of gold or plastic material insert a plug so perfectly, that while possessing uniform density, it shall exclude moisture from filtering in at any point between itself and the parieties of the cavity. And where these conditions are fulfilled, a perfect plug inserted into sound dentine, and with no roughness or concavities for the retention of putrescent organic matter, the destructive process is arrested for an indefinite time. It is here that the necessity for special training makes itself evident. The carious matter must be carefully removed, without recklessly penetrating the cavitas pulpæ, and the cavity must receive such a form as to retain the plug, which again must be so deftly inserted, that it shall offer perfect occlusion without flaw and without undue pressure on the sensative structure within. It will easily be understood that there are various subsidiary matters, which want of time and the fear of exhausting your patience forbid me to touch upon, connected with the insertion of the plug or stopping; such as maintaining a dry state of the cavity and of the several portions of gold which have to be welded together, a good light in which to operate, and a chair so constructed as to place the patient in the required position without discomfort. Neither may we stop to enquire how it is that the living dentine can be made so tolerant of the close impaction of inorganic matter; or how it is that the different rate of expansion under varying temperature in the tooth-bone and the metallic plug, to say nothing of the high conducting power of the latter as regards heat and cold, does not lead to disastrous consequences. Suffice it to say, that notwithstanding these contingencies, a very large measure of success is obtained by the operation known as stopping teeth, by which, as is probable within the experience of almost all here present, they are only prevented from becoming sources of agonizing and prostrating pain, but are made valuable aids to digestion, to health, and to the prolongation of life. Nor is it necessary to take account of complications arising from abnormal sensibility, whether of the dentine or of the exposed pulp, or of the minute attention to details of apparently minor importance, but not less essential to a successful result, to show that Dental surgery can only be taught and practiced as a special branch, and by technical professors. The case adduced in illustration, is one of everyday occurance, and whether regarded in reference to the manipulative skill required, or the time occupied, which may vary from half-an-hour to five or six, or even more hours, makes it quite incompatible with ordinary surgical practice.

Concurrently with a rapid and brilliant advance in the science and art of our specialty, due to a large extent, to a wave of progress which reached this country from the other side of the Atlantic, there arose an increasing demand for, and appreciation of, its services on the part of the public. And hence the necessity for an organization of the profession forced itself upon the attention of those who might be supposed to feel an interest in its welfare. On such an important matter it was hardly to be expected that unanimity would prevail, and accordingly it soon became evident that there were two competing schemes, by one of which the Surgical diploma was regarded as imperative and sufficient, while it was repudiated by the other as being of little worth and relevance.

What seemed certain was that neither alternative was adapted to meet the exigencies of the case; for although the possession of the diploma of the Royal College of Surgeons was felt to ensure a good social and educational standing, and so far to be advantageous, it could not be regarded as giving evidence of that special knowledge which has been shown to be necessary, inasmuch as the subject found no place in the examinations. On the other hand, to sever the connection of the specialty with general surgery, by the establishment of a College of Dentists, was regarded as a derogation, neither necessary nor desirable, from the position which it had hitherto enjoyed. Under these circumstances, it was resolved to memorialize the Royal College of Surgeons on the subject, and to ask for a special diploma or license to be granted on examination by a conjoint board consisting of half surgeons and half specialists, and for such modification of the prescribed course of study as should eliminate what was valueless and substitute what would best meet the requirements of the case; so that in the result, the educational standard should not be so much lowered as varied. And it was found, practically, that the points of contact were so much more numerous than those of divergence in the lines of study for the diploma for general surgery and for the licentiateship of dental surgery respectively, that a yearly increasing number of dental students has set the good example of possessing themselves of both qualifications. Nevertheless, it will still be found both convenient and advantageous that the surgical treatment of diseases of the maxillæ or of the soft structures in connection with the oral cavity, shall be confided to the practised hands of the pure surgeon. To borrow an illustration from the sister art of music, it would not be reasonable to expect that he who stirs our soul with the sonorous thunders of the organ should equally be able to hold us spell-bound with the "linked sweetness long drawn out" of the violin. An acquaintance with the science and theory of music must be the basis of proficiency in both cases, but the attainment of high excellence in either can only be the reward of close and daily devotion to the selected one. So in the case in question, a knowledge of anatomy, physiology, and pathology is necessary as a common and firm ground in consultation, and as an aid in diagnosis in the protean forms of facial neuralgia. It is to the practised eye of the specialist that the insidious caries reveals itself before it becomes patent to the ordinary observer, and thus is the patient spared much suffering and much useless constitutional treatment. Or he may be able to refer the attack to exostosis, to malformation, to malposition, arrest of development, or retarded eruption of any particular tooth, with equally good effect. Such knowledge is also of conspicuous advantage in cases of hemorrhage after extraction, whether traumatic or due to hemorrhagic diathesis. in the management of anæsthetics, or in the more modern operation of replantation, in which, strictly following the lines of antiseptic treatment---teeth in a state of partial necrosis, and giving rise to suppuration, are removed, and after excision of the necrosed parts, are re-inserted in their alveoli.

Great, however, as was the advantage of the institution of the licentiateship of dental surgery, it was soon found to be an incomplete work, inasmuch as the powers conferred by it were of an enabling but not of a compelling kind. For the first time, indeed, in the history of the profession, a special institution was provided for the treatment of the diseases of the teeth on a sufficiently comprehensive scale; and also a school of dental surgery, where instruction was given by lectures and by clinical teaching, in accordance with a curriculum determined upon by the dental examining board. But although advantage was taken of these facilities to a large extent by those who were anxious to practise their profession with credit to themselves and advantage to the public, yet in the absence of a register there was still no check to the intrusion of the unprincipled and uninstructed. This has now been happily accomplished by the Dentists Act of 1878, introduced by Sir John Lubbock, which forbids, under penalties, the use of the word Dentist, or any other title, by any one, implying that he is qualified to practise dental surgery, unless his name appears on the Register. And although, in its extreme solicitude for vested interests, the legislature made it possible for a large number who had little or no ground to claim a place on the Register, to enrol themselves prior to August 1st, 1879, when the Act should take effect; yet the fact stands, that only those who by a well-considered course of study, and by examination, shall have proved themselves duly qualified to practise the specialty, can henceforth be permitted to enter its ranks. This is what has been accomplished. Legislative sanction has been obtained for a scheme not directed to give prominence to the educated and qualified few, but to raise the whole body of the profession; not to accentuate the distinctive character of the speciality, but indissolubly to unite it to the great surgical body, through the examining board of the Royal College of Surgeons. And if, by reason of legal technicalities and definitions, not always in harmony with the common understanding, the Register must still be encumbered with a large number of ineligible and undesirable names, consolation must be sought for what has not been accomplished, in the reflection that time, which tries all things, will surely, though it may be slowly, redress this wrong.

Such is the present position of a branch of surgery which, within living memory, has received a very marked and rapid development, and which is destined to play no unimportant role in the cycle of medical science, which has counted among its foremost practitioners men who would have won distinction in any calling: Fox, Bell, Cartwright, Nasmyth, Rogers, Tomes, and many others, who were distinguished for their energy and skill in operations, or who have enriched the literature of the specialty by monographs, or by treatises of a scientific and practical character. Nor must it be forgotten that in its scientific aspect it did not fail to engage the great and original mind of John Hunter; and coming down to later times, that it possesses a cherished treasure in the Odontography of Professor Owen. Nor, lastly, must we ever fail to remember with feelings of the deepest gratitude that it was in the congenial soil of this specialty that that flower of sweetest perfume and of loveliest form, Anæthesia,* arose to soothe and bless the sense and heart of man.

* The first surgical operation under induced insensibility to pain was the extraction of a tooth under the influence of nitrous oxide or laughing gas.

